# Customized z-shaped versus conventional miniplates for fixation of parasymphyseal/body mandibular fractures: a randomized controlled trial evaluating clinical and radiographic outcomes

**DOI:** 10.1186/s12903-026-07671-6

**Published:** 2026-02-02

**Authors:** Mariam A. Abd EL Hamid, Ahmed S. EL Mahallawy, Nehad M. Elshatby, Mohamed E. Saber

**Affiliations:** 1 Oral and Maxillofacial Department, Faculty Of Dentistry, Alexandria University, Champollion St., Azarita, Alexandria, 21527 Egypt; 2 Oral and Maxillofacial Surgery Department, Faculty of Dentistry, Alexandria University, Alexandria, Egypt; 3 Faculty of Medicine, Alexandria University, Alexandria, Egypt

**Keywords:** Customized z-shaped miniplate, Conventional two miniplates, Mandibular fractures

## Abstract

**Background:**

Mandible is considered the protrudest mobile bone of the facial skeleton so it is the most common maxillofacial fracture. Customized Z miniplate is a new geometry that may provide better mental nerve preservation, healing of fractures compared with conventional two miniplates in the treatment of parasymphyseal/body mandibular fractures.

**Aim of the study:**

To compare between customized Z miniplates and conventional miniplates for fixation of parasymphyseal and body mandibular fractures in terms of bone density (primary outcome), post operative mental nerve paresthesia, pain, occlusion and operation time (secondary outcomes).

**Materials and methods:**

This prospective, parallel, exploratory, randomized controlled study was conducted from April to December 2024 and included 22 patients diagnosed with parasymphyseal or body mandibular fractures. Allocation (customized Z-miniplates, *n* = 11; two miniplates, *n* = 11) was done by independent statistician using a computer-generated random number list method. Mann-Whitney U, Wilcoxon-signed rank and Chi-square tests were applied (*p* < 0.05). Postoperative patient evaluation was done for primary outcome (bone density) and for secondary outcomes (nerve paresthesia, postoperative pain, occlusion and operation time). All adverse events were recorded prospectively during follow-up visits. The trial was registered retrospectively at ClinicalTrials.gov (NCT07094867) due to administrative procedures, which represents a potential source of bias.

**Results:**

Statistical analysis did not detect significant variation in terms of mean bone density, occlusion, mental nerve affection and pain between customized Z miniplates and conventional two miniplates. Operation time was statistically significant shorter in customized Z miniplate group (*p* < 0.001). Only one case of wound dehiscence occurred in each group and no other complications observed.

**Conclusion:**

Both fixation methods provided results that were not statistically distinct in terms of pain, occlusion, mental nerve affection and mean bone density, with significant shorter operation time for parasymphyseal/body mandibular fracture fixation. Given the sample size, our study provides preliminary findings of clinical and radiographical performance of customized Z plate.

**Trial registration:**

The research was formally recorded in the clinicaltrials.gov database with registration submitted on 06/27/2025 under the number (NCT07094867).

**Supplementary Information:**

The online version contains supplementary material available at 10.1186/s12903-026-07671-6.

## Introduction

Mandible is considered the largest and strongest facial bone. The incidence of mandibular fractures ranges between 40 and 62% [1, 2]. Fractures at the transition zone between the parasymphyseal/body compromise about 9% − 57% of all mandibular fractures [3].

The common causes of mandibular fractures are road traffic accidents and alleged assault. Other mode of traumas is fall from heights, sports-related injuries, and occupational-related accidents [4].

Mandibular fracture management has been evolved over years. In 1973, Michelet et al. introduced a bendable, non-compression mini plate placed through intra-oral approach and fixed with mono-cortical screws [5]. Nowadays, primary intervention for mandibular fractures includes either closed reduction (with or without intermaxillary fixation) or open reduction and internal fixation (ORIF) by plates and screws [6]. 

Although conventional miniplates show adequate stability for the fractured segments, However, higher complication rates particularly paresthesia of the mental nerve could be happened [7]. With better understanding of mandibular biomechanics, different plate designs and modifications have been proposed to improve the clinical results [6]. 

Farmand and Dupoirieux [8] developed three-dimensional plates based on the concept that a quadrangular shape has stable configuration that give good stability for fixation. However, multiple studies in the literature noted that 3D miniplate system is challenging in its adaptation and hard to be used at fractures involving mental nerve often requiring complex bending.

To overcome the limitations of both the conventional two-miniplate system and the 3D plate, Prajwalit introduced a Z-shaped miniplate design, a new geometry of miniplates for fixation of parasymhyseal/body mandibular fractures, intended to offer improved stability, easier adaptation to the mandible, and preservation of the mental nerve. The oblique connected bar is proposed to improve strength and cross-stabilization, while the open-ended design allows for less traumatically plate placement near fracture segments involving mental nerve [9]. 

Computer-aided design and computer-aided manufacturing (CAD/CAM) plate design is seeking maximum reduction volume on an original palate resulting in less operation time, better stability of the fixation system & decrease the possibility for plate removal [10, 11].

Customized fixation mechanism provides more plate adaptation to the bone surface without distortion so decrease the time needed to bend and fix the plate intra-operatively and meet the criteria of a semirigid fixation with low complication rate compared with standard miniplates [9, 12].

Recent randomized and comparative studies have evaluated patient-specific and 3D-printed fixation versus conventional miniplates in mandibular fracture fixation, concluding improved plate adaptation to bone and, in some series, reduced operative time with customized devices [13–15]. Systematic reviews have begun to make use of this evidence and highlight benefits of CAD/CAM workflows [16, 17] These findings strengthening the present study that compare between customized Z-miniplates versus conventional two-plate fixation.

The gap of knowledge is that there is a shortage of clinical studies comparing between different geometries of the miniplates to give knowledge about the best option for treatment of parasymhyseal/body mandibular fractures.

The aim of this study is comparing between customized Z miniplates and conventional two miniplates in the fixation of parasymphyseal and body mandibular fractures in terms of post operative mental nerve paresthesia, pain, occlusion and bone density.

The null hypothesis is there is no significant difference between customized Z shaped miniplate and conventional 2 miniplates in the treatment of parasymhyseal/body mandibular fractures in terms of post operative mental nerve paresthesia, pain, occlusion and bone density.

## Materials & method

Patients with parasymphyseal or body mandibular fractures indicated for surgery were recruited prospectively between April and December 2024 from cases admitted to the Emergency Department at Alexandria University Teaching Hospital. All patients were operated on and followed up at the Oral and Maxillofacial Surgery Department, Faculty of Dentistry, Alexandria University, Egypt.

Primary outcome was to measure the mean bone density at the site of the fracture between study and control groups, while secondary outcomes were to assess post operative pain, occlusion stability, sensory nerve affection and operation time between 2 groups. The study protocol was established prior to patient enrollment and all reported outcomes were totally adhered to the original protocol, not modified after recruitment began.

Bite force measurements were not included in this study due to the unavailability of a bite force device at our facility, which would have required collaboration with the Faculty of Engineering.

Sample size was calculated assuming an 80% power and 95% confidence interval (CI) to assess the difference in bone density between patients treated with custom-made Z pattern plates and standard 2 miniplates. Dessoky et al. [18] reported the mean Standard deviation (SD) bone density in patients who were treated with custom-made plates at 3 months was 900.4 (148.55). Harby et al. [19] reported the mean (SD) bone density in patients treated by standard 2 miniplates at 3 months was 952.8 (53.40). The calculated 95% CI = (−73.56, 178.36) and pooled SD = 119.92. Assuming that the effect size = 1.35, the minimum required sample size was calculated to be 10 patients, increased to 11 to make up for the 10% loss to follow up. The total required sample size = 2 × 11 = 22 patients [20]. Sample size calculated by MedCalc Statistical Software version 19.0.5 (MedCalc Software bvba, Ostend, Belgium; https://www.medcalc.org; 2019).

This study was powered based on the single primary outcome (bone density). All other outcomes including postoperative pain, sensory nerve function, occlusion stability and operation time were considered secondary outcomes that were not included in the original power calculation; therefore, no formal multiplicity adjustment was stablished. So, secondary outcomes analyses were presented as exploratory and are interpreted with emphasis on effect sizes and 95% confidence intervals rather than statistical significance alone.

### Eligibility criteria and study design

Inclusion criteria included adults (20–42 years) dentulous, of both genders, medically fit (ASA I) with unilateral parasymphyseal/body mandibular fractures (to maintain homogeneity in fracture pattern and surgical technique) presented with disturbed occlusion and accepted the digital surgery to ensure ethical compliance and patient understanding all workflow. Exclusion criteria included patients with comminuted, pathological, infected, non-displaced fractures and patients with known allergies.

**Fig. 1:**
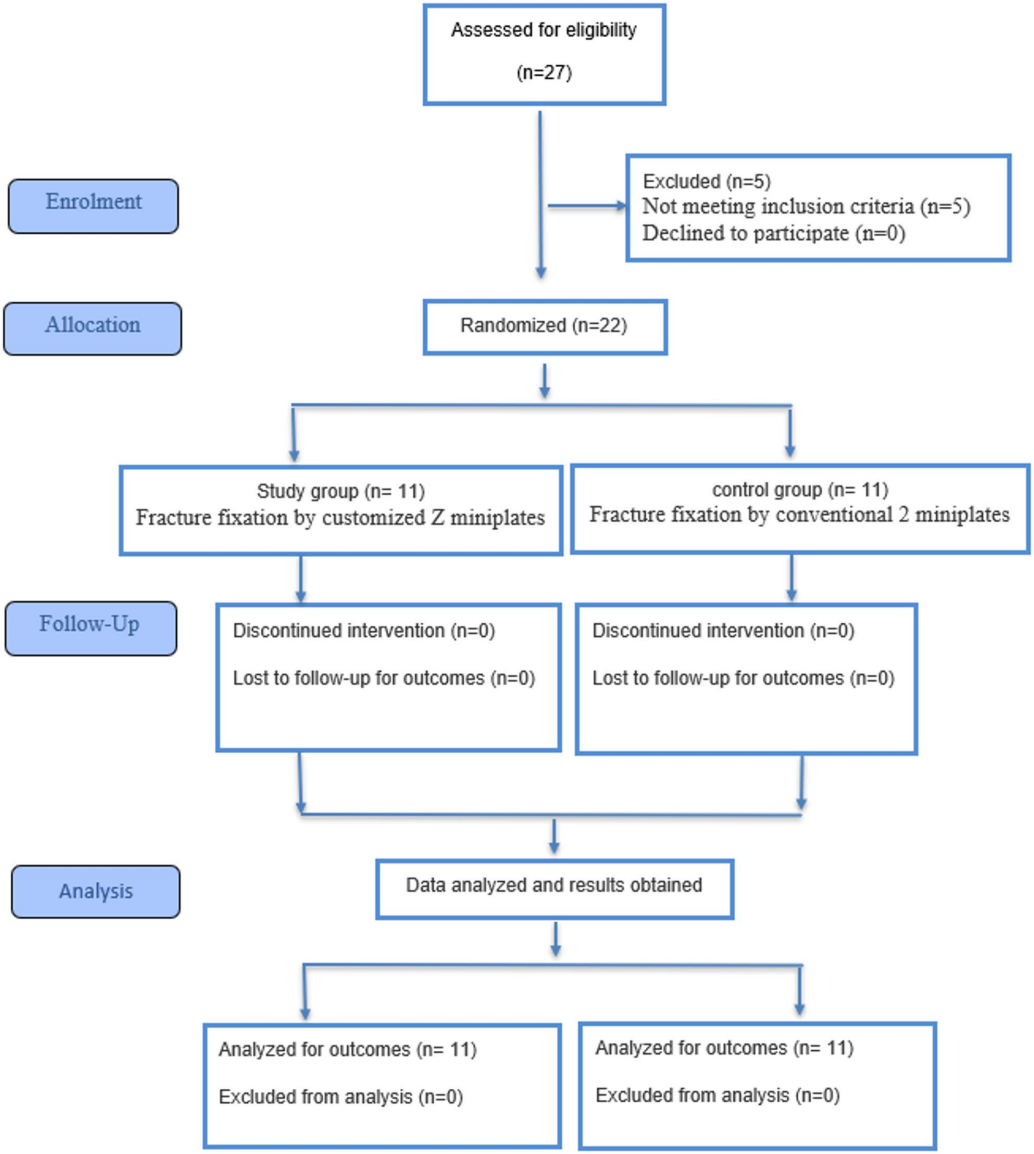
CONSORT Flow diagram

This prospective parallel, exploratory, randomized controlled clinical trial with a 1:1 allocation ratio and was reported according to the CONSORT guidelines. 22 patients were enrolled, with 11 patients allocated to the customized Z-miniplate (study) group and 11 to the conventional miniplate (control) group. (Fig. 1**)**

### Ethical compliance and registration

The study was conducted in accordance with the Helsinki Declaration (2013) and approved by the research Ethics Committee of Alexandria University Faculty of Dentistry, Egypt (IRB No. 001056 – IORG 0008839 with ethics no. 0866-02/2024) prior to any research-related activities. Informed consent was obtained from each patient prior to their enrollment and any surgical intervention after explaining the study objectives and procedures.

This study is retrospectively registered at clinicaltrials.gov (NCT07094867), with registration submitted on 27 June 2025. The first participant was enrolled on 20 April 2024 [1st operation on 22/4/2024 and last operation on 19/12/2024]. Retrospective registration occurred due to administrative and institutional procedures. The full trial protocol and statistical analysis plan are publicly accessible on ClinicalTrials.gov. The study protocol, eligibility criteria, randomization and primary outcome measures were prespecified before first patient enrollment and were not changed throughout the trial in accordance with CONSORT guidelines. Nevertheless, retrospective registration represents a potential source of bias.

### Randomization and allocation

Patients were divided into two groups based on treatment type using block randomization of block size 4. An independent statistician generated the random sequence using a computer-generated random number list method by (https://www.randomizer.org/). To ensure allocation concealment, the same statistician prepared sealed opaque sequentially numbered and tamper-proof envelopes which were stored in secure location. After patient enrollment and completion of baseline assessment, an independent coordinator (not involved in surgery or data analysis) opened the envelope to allow digital planning and milling process. This protocol ensured that the researchers enrolling participants were blinded to the next assignment until the moment of allocation.

### Blinding and bias mitigation

Sensory nerve function and pain assessment were performed by a consultant of rheumatology, rehabilitation and Physical Medicine with more than 15 years of experience in neurophysiology, who was not a member of the surgical procedures and was blinded to group allocation. To enhance intra-observer reliability, all assessments were recorded using a standardized protocol and repeated twice by the same blinded consultant.

Blinding was not feasible for assessments of bone density, occlusion stability and operation time, due to the visibility of plate type on CT images. To mitigate potential detection bias, we implemented several strategies: First, while measurements were recorded by two assessors prospectively of surgical team demonstrating excellent intra and inter-examiner reliability with very high ICC values (≥ 0.99) and narrow confidence intervals close to 1.0, the final statistical analysis was performed by a statistician who was blinded to intervention till analysis was complete and received data in coded form. Second, HU measurements were done using a pre-specified, standardized protocol for ROI placement. Third, CT scans were presented to the assessors in a randomized order, stripped of patient identifiers and group labels, to minimize expectation bias. While these measures strengthen the objectivity of the findings, the lack of full radiological blinding is acknowledged as a limitation.

### Materials

**Fig. 2:**
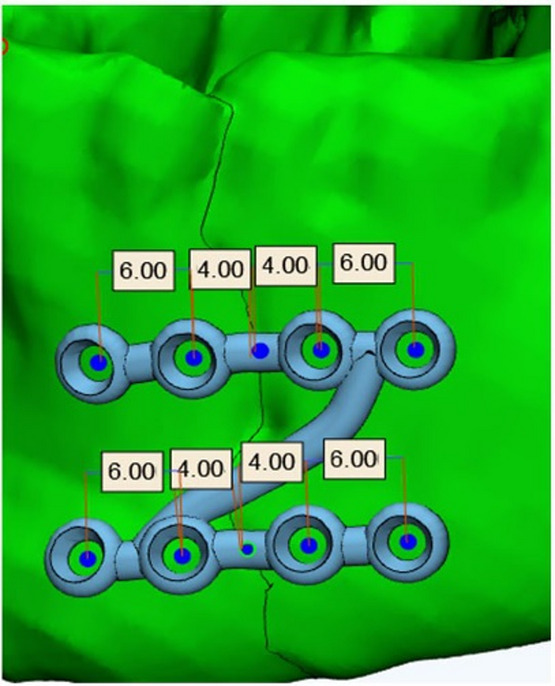
3D planning of the customed Z plate

1. Customized Z shaped titanium miniplate (https://www.tiint.com/): composed of 2 horizontal arms connected by an oblique bar (1.2 mm thickness to ensure adequate biomechanical stability) designed with 4 nonlocking screws holes (2mm diameter), inter-screw hole distance crossing fracture line is 8 mm while the distance between the 2 holes at each side of fracture line with is 4 mm. (Fig.2) The oblique bar length was customized depending on patient-specific mandibular length and contour to ensure adaptation and mental nerve avoidance (taking into consideration dental root injury avoidance).
2. Standard titanium (https://www.tiint.com/) miniplates.
3. Titanium screws 7- and 9-mm length & 2 mm head diameter.
4. Mimics 21.0 software (Materialise Software, Leuven, Belgium).
5. 3-matic Research 13.0 (x64) software.

#### Presurgical phase: Virtual Planning

##### CT acquisition

All CT scans were performed using the same Philips 16-detector CT scanner at baseline and at 6 months. CT parameters were standardized and matched for each patient’s follow up: tube voltage 100–130 kVp (depending on patient weight), tube current 150–175 mAs, slice thickness 1 mm, and images were reconstructed using a high-resolution bone kernel. To ensure measurement reliability and avoid metal artifacts, ROIs were placed 2 mm away from hardware by two independent assessors. Daily automated air/water calibrations were performed by the CT scanner’s internal quality assurance system to ensure the stability and accuracy of HU measurements throughout the study period.

##### Virtual planning (Study group)

**Fig. 3:**
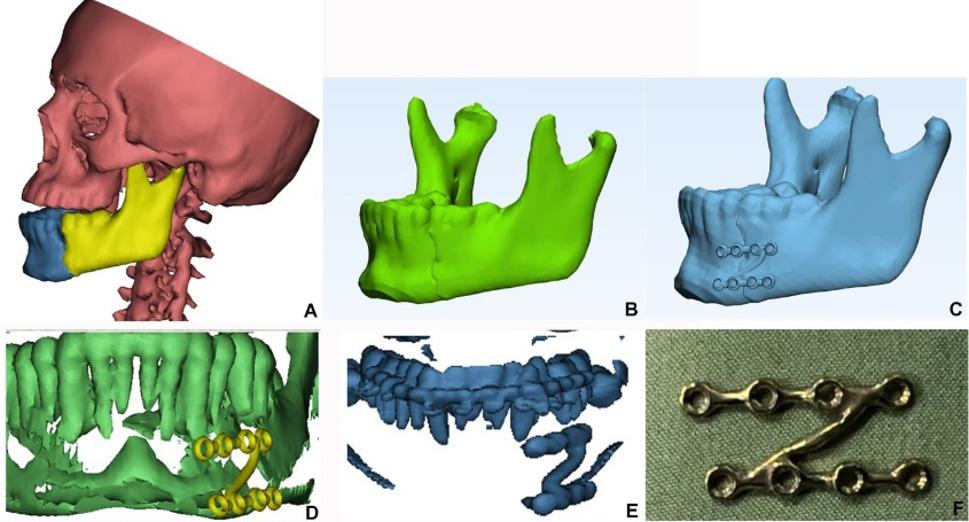
**A**) 3D reconstruction of skull and fractured segments of mandible, **B**) Digital reduction and union of fractured segment, **C**) Digital planning of customized Z plate, **D**) Root injury avoidance was checked preoperatively and, **E**) postoperative 3D reconstructed mandible after reduction and fixation by customized Z- shaped miniplate revealed that upper arm of plate is away from teeth roots **F**) plate after milling

CT DICOM file were imported into Mimics to create 3D reconstruction of skull and mandible, proximal and distal of mandible were segments separately, then transfer at 3-matic. The fractured segments were reduced by mirroring the healthy side of mandible. The patient specific Z-plate was designed**)**. Overlapping the plate design on 3D of mandible at Mimics Research 21.0 was done to avoid root injury. (Fig. 3A-E) Final STL format of plate was sent for milling on a titanium block. Sterilization of milled plate (Fig. 3F).

To ensure accuracy, the final virtual reduction was validated by condylar position within glenoid fossa, occlusion (no tooth intersection after reduction) and symmetry (validation was performed through mirroring of both contralateral side of the mandible and skull with deviation values range from 0.001 to 0.004).

##### Virtual Planning (Control group)

The virtual reduction of mandibular fracture was done in the same process as study group to obtain a virtual reduced mandible.

#### Surgical procedure

All patient received necessary preoperative analgesics and prophylactic antibiotic and were treated under general anesthesia. Maxilla-Mandibular Fixation (MMF) was applied following fracture exposure intra-orally and guided by patient occlusion.

- For group A: customized Z plate was fixed after confirming its adaptability to bone and avoidance of mental nerve pressure.
- For group B: 2 miniplates adaptation and fixed according to Champy’s osteosynthesis lines.
- The operation time was recorded from reduction to fixation only for all of the cases. (Notinvolving preoperative time needed for virtual planning or MMF placement.)
- Standardized postoperative care, including ice pack application, prescribed medication andoral hygiene instruction for all patients.

#### Follow up phase

Patients in both groups were followed up at 24-hours, one, four, six, and twelve weeks postoperatively to assess this parameter:

1. Pain postoperatively was recorded by 10-point Visual Analogue Scale (VAS) [22].
2. Postoperative occlusion was checked by adequate occlusion including centralization of midline, canine and molar relationship.
3. Sensory nerve function was tested subjectively (patient report), objectively (Dental probe comparison) [23] and Electrophysiologically using the TruTrace DeyMed system for all patients examined in a supine position with standardized settings (pulses of 0.2 ms duration, and stimulus intensity 10–20 mA, adjusted for each patient tolerance. This test evaluates sensory amplitude, nerve conduction velocity (NCV), and latency comparing healthy and fractured sides. Bipolar sensory stimulator was applied over mental foramenarea and Sensory nerve action potentials (SNAPs) were recorded using reusable surface electrodes (active electrode placed 3 cm in front of tragus below zygomatic arch, referenceelectrode placed above zygomatic arch and the ground electrode placed on forehead).Electrode strength was kept below 5 kΩ. We relied on side-to-side amplitude comparison, which is a validated principle in electrodiagnosis to indicate axonal injury [24, 25]. The primary neurosensory outcome was defined as a binary measure (presence or absence of paresthesia) based on patient reported symptoms. Subjective evaluation was confirmed by objective clinical probe testing (light touch and pin-prick). For electrophysiologicalassessment, a side-to-side comparison was done, a nerve was considered injured when the SNAP amplitude was reduced to less than 50% of the contralateral healthy side. All recordings were standardized regarding equipment, electrode placement and environmental conditions, and were performed by a consultant blinded to the surgical groups.

**Fig. 4:**
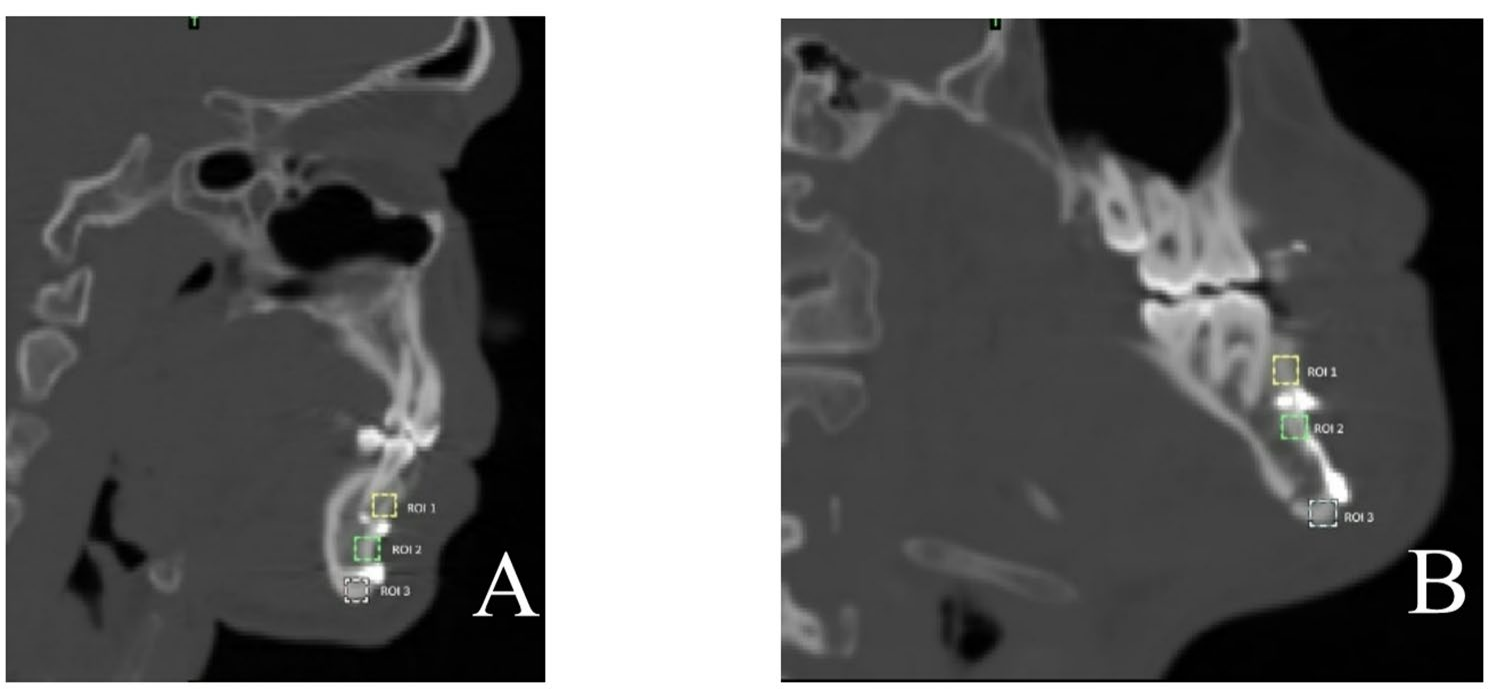
ROI diagram. Standardized Three fixed-size squares placement for bone density measurements along the fracture line on sagittal CT cuts. **A** Conventional 2 miniplates (1st below the lower plate, 2nd at the middle of fracture line between 2 plates and 3rd above the upper plate). **B** Customized Z miniplate (1st below the lower plate, 2nd just below the upper arm above oblique arm and 3rd just above the upper plate)

Radiographically, mean bone density was measured by Mimics at fracture site on sagittal CT cuts (immediate post operative and post 6 months CT) by placing three standardized Regions of Interest (ROIs) which are fixed squares along the fracture line. Regarding conventional miniplates, ROIs were placed below the lower plate, at the middle of fracture line between plates and above the upper plate). Regarding customized Z miniplate, ROIs were placed below the lower plate, just below the upper arm above oblique arm and just above the upper plate. (Fig. 4)

The Hounsfield Unit (HU) at 6 months was selected as the primary surrogate endpoint as it provides a quantitative and objective scale to evaluate the mineral density and structural integrity of the newly formed bone, representing higher sensitivity in detecting subtle differences in the quality of bone regeneration between the two groups.

All adverse events were recorded prospectively during follow-up visits, including infection, wound dehiscence, plate complications, need for plate removal, occlusal changes, and any radiation-related issues due to repeat imaging.

#### Statistical analysis

Normality of the continuous data was tested using the Kolmogrov-Smirnov and Shapiro-Wilk tests. Continuous data were described by the mean, standard deviation (SD), median, interquartile range (IQR), median difference, minimum, and maximum values. Categorical data were described by frequency and percentage. Differences between Customized Z plates and the conventional 2 miniplates in associated fracture (other body fractures), fracture site, mode of trauma, and occlusion were assessed by Chi-squared test with Fisher’s exact or Monte-Carlo corrections. Differences between Customized Z plates and the conventional 2 miniplates in bone density and operation time were assessed by Mann-Whitney U test. Difference in bone density in each type of plates was assessed by Wilcoxon-signed rank.

For changes in pain VAS, between-group comparisons at individual time points were conducted using the Mann–Whitney U test. Within-group changes over time were assessed using the Friedman test, followed by Bonferroni post-hoc adjustment. A generalized estimating equation (GEE) with Gaussian distribution and identity link was fitted to model population-averaged effects, using robust standard errors. Assumptions of the linear GEE model were assessed descriptively. Independence between clusters was ensured by design, and time was modeled as a categorical factor to avoid imposing linearity. An exchangeable working correlation structure was specified, and time points showing no variability across participants (preoperatively, at 6 weeks, and 12 weeks follow up) were excluded from the model. Linearity of continuous predictors and residual behavior were evaluated visually, with no major violations observed. Although VAS scores are bounded (0–10), which can theoretically violate the normality assumption, robust standard errors were used to ensure valid inference. Histograms of residuals did not indicate extreme deviations from symmetry, and predicted values remained within the plausible range. Certain time points were excluded from the GEE model for conceptual reasons. The preoperative measurement (baseline, all values = 10) reflects a uniform starting point and does not differentiate participants. The 6-week (all values = 1) and 12-week (all values = 0) measurements represent terminal stages in which the outcome was uniform across participants. As no meaningful between-group or within-subject differences were possible at these stages, longitudinal modeling was restricted to the earlier, analytically informative phase (postoperative, 1-week, and 4-week time points).

For sensory nerve subjective and objective testing, the difference between the plates at each time point was assessed by Chi-squared test with Fisher’s exact test. Difference within each group over time was tested by Cochrane Q test. A GEE model with a binomial distribution and logit link was fitted, using robust SEs. The 6-week and 12-week time points were excluded from the model because all participants had uniform outcomes at these stages, representing the terminal phase of recovery. The 1-week and 4-week assessments were collapsed into a single “early follow-up” category to reflect the early recovery phase of postoperative nerve recovery (If a patient had “Yes” at either time, the collapsed value was coded as “Yes”). Models’ results were reported using the Wald χ², degree of freedom (df), standard errors (SE), 95% confidence intervals (CI), estimated marginal means, or adjusted odds ratios (Exp B). Between-group comparisons of categorical outcomes at individual time points were conducted using Chi-square tests. Within-group changes over time were assessed using the McNemar test.

Effect sizes, test statistics, and 95%CI were calculated. Significance level was set a *p* < 0.05. Analysis of data was done using SPSS version 23.0 for Windows (SPSS Inc., Chicago, USA).

All analyses were conducted on an intention-to-treat analysis (ITT), as all patients completed the study according to the prespecified protocol without any deviations or loss to follow-up.

## Results

After follow up completion, all patients allocated to the study completed the protocol with no loss and no protocol deviations occurred. Total 22 patients were recruited in the study involving 7 females and 15 males. Ages ranged between 20 and 42 years with mean 27.55 ± 6.68 at study group and mean 29.09 ± 8.24 at control group. Causes of the trauma were road traffic accidents (*n* = 18) with mean 90.91% at study group and mean 72.73% at control group, Inter personal Violence (*n* = 1) with mean 9.09% at control group and falling from heights (*n* = 3) with mean 9.09% at study group and mean 18.18% at control group. (Table 1)

There is no statistically significant difference between 2 groups in terms of preoperative mental nerve paraesthesia, severe fracture displacement, no history of smoking and surgery performed within 48 h of trauma.

**Table 1:** Description of the study sample (*N* = 22)^b^

Variables		Customized Z plate *n* = 11	Conventional 2 miniplates *n* = 11	*p* value
Age	Mean (SD)	27.55 (6.68)	29.09 (8.24)	0.63^a^
	Median (IQR)	28.00 (20.00, 33.00)	28.00 (21.00, 37.00)	
	Min-Max	20.00–40.00	20.00–42.00	
Gender	Females N (%)	3 (27.27)	4 (36.36)	1.00^b^
	Males N (%)	8 (72.73)	7 (63.64)	
Associated fracture	Yes N (%)	4 (36.36)	5 (45.45)	1.00^b^
	No N (%)	7 (63.64)	6 (54.55)	
Fracture site	Body of mandible N (%)	7 (63.64)	6 (54.55)	1.00^b^
	Parasymphyseal N (%)	4 (36.36)	5 (45.45)	
Mode of trauma	Road traffic accident N (%)	10 (90.91)	8 (72.73)	0.46^c^
	Fall N (%)	1 (9.09)	2 (18.18)	
	Interpersonal violence N (%)	0 (0.00)	1 (9.09)	

*SD* Standard deviation, *IQR* Interquartile range, *Min* Minimum, *Max* Maximum

^a^Mann-Whitney U test, ^b^ Chi-square with Fisher’s-exact correction, ^c^ Chi-square with Monte-Carlo correction

^b^Variables with no variability (100%/0%) across both groups are not shown as they do not contribute comparative value

### Clinical evaluation data

All surgical procedures in both groups were done within 48 h of trauma. In the study group, the digital workflow (including virtual planning and plate fabrication) was completed within this timeframe, and no delays to surgery were recorded due to the customization process.

Regarding the assessment of mental nerve function, the subjective and objective tests revealed significant improvement in sensation across the follow up period for 2 groups p-value < 0.001. For the subjective test, the effect size was notably high at 1.00 during the 1 st 4th weeks intervals, indicating a strong correlation between time and sensory recovery. Similarly, objective testing demonstrated a consistent recovery pattern with a maximum effect size of 1.00 at 1, 4, 6, and 12 weeks. At the final 12-week follow-up, 100% of patients in both groups achieved complete sensory recovery (Table 2) Results of GEE models are at supplementary files (Table 2’, 2’’, 2’’’).

**Table 2:** Difference between customized z plates and conventional 2 miniplates in postoperative sensory nerve function measured by the subjective and objective tests (N=22)

Electrophysiological test			Customized Z plate n = 11	Conventional 2 miniplates n = 11	Median difference	Z	Effect size	95% cI	*p* value ^a^
Amplitude (µV)	Fractured	Mean (SD)	15.35 (4.05)	14.55 (3.48)	−0.40	−0.16	0.03	(−4.20, 2.80)	0.90
		Median (IQR)	14.40 (13.00, 18.20)	18.00 (14.30, 20.90)					
		Min-Max	10.40–23.00	9.80–21.00					
	Sound	Mean (SD)	18.27 (3.92)	18.65 (3.41)	0.50	−0.49	0.10	(−3.00, 4.20)	0.65
		Median (IQR)	15.00 (10.50, 16.80)	20.20 (14.90, 21.00)					
		Min-Max	13.50–25.00	13.00 −23.00					
Median difference			2.50	4.00	-				
Z			−2.94	−2.93					
Standardized effect size			0.63	0.62					
95% CI			(1.70, 4.50)	(2.80, 5.35)					
*p* value^b^			0.003*	0.003*					
Latency (ms)	Fractured	Mean (SD)	2.23 (0.70)	2.49 (1.16)	−0.10	−0.56	0.12	(−0.40, 0.80)	0.61
		Median (IQR)	2.10 (2.00, 2.30)	2.00 (1.70, 4.10)					
		Min-Max	1.40–4.20	1.40–4.40					
	Sound	Mean (SD)	2.24 (0.68)	2.69 (1.15)	0.20	−0.96	0.20	(−0.20, 0.80)	0.37
		Median (IQR)	2.00 (1.80, 2.50)	2.20 (2.00, 4.00)					
		Min-Max	1.60–4.00	1.70–4.80					
Median difference			0.00	0.20	-				
Z			−0.04	−1.71					
Standardized effect size			0.01	0.36					
95% CI			(−0.20, 0.25)	(0.00, 0.45)					
*p* value^b^			0.96	0.09					
Conduction velocity (m/s)	Fractured	Mean (SD)	43.09 (2.12)	43.82 (2.40)	−1.00	−0.56	0.12	(−1.00, 3.00)	0.61
		Median (IQR)	43.00 (41.00, 45.00)	44.00 (42.00, 47.00)					
		Min-Max	40.00–46.00	41.00–49.00					
	Sound	Mean (SD)	44.55 (2.73)	43.73 (3.20)	−1.00	−0.73	0.16	(−4.00, 2.00)	0.48
		Median (IQR)	44.00 (42.00, 47.00)	43.00 (41.00, 47.00)					
		Min-Max	41.00–49.00	40.00–49.00					
Median difference			2.00	0.00	-				
Z			−1.66	−0.04					
Standardized effect size			0.35	0.01					
95% CI			(−0.50, 3.00)	(−2.50, 2.00)					
*p* value ^b^			0.10	0.96					

^a^Chi-squared test with Fisher’s Exact correction, ^b^Cochrane Q test

*Statistically significant at *p *< 0.05

Regarding the electrophysiological study, the mean value of amplitudes of the healthy side at study group was18.27 ± 3.92 µV and at control group was18.65 ± 3.41 µV, while the mean value of amplitudes of the fractured side at study group was 15.35 ± 4.05 µV and at control group was mean value of 14.55 ± 3.48 µV. A statistically significant difference was detected between the healthy and fractured sides in 2 groups in amplitude (p-value = 0.003). In study group, comparison between fractured and sound sides showed effect size of 0.63 with CI (1.70, 4.50) and in control group, effect size was 0.62 with CI (2.80, 5.35). Results indicates that improvements in nerve function were statistically reliable both fixation methods provided a conducive to nerve recovery without significant interference (Table 3).

**Table 3:** Difference between customized z plates and conventional 2 miniplates in Sensory nerve function measured by electrophysiological test (N=22)

Electrophysiological test			Customized Z plate n = 11	Conventional 2 miniplates n = 11	Median difference	Z	Effect size	95% cI	*p* value ^a^
Amplitude (µV)	Fractured	Mean (SD)	15.35 (4.05)	14.55 (3.48)	−0.40	−0.16	0.03	(−4.20, 2.80)	0.90
		Median (IQR)	14.40 (13.00, 18.20)	18.00 (14.30, 20.90)					
		Min-Max	10.40–23.00	9.80–21.00					
	Sound	Mean (SD)	18.27 (3.92)	18.65 (3.41)	0.50	−0.49	0.10	(−3.00, 4.20)	0.65
		Median (IQR)	15.00 (10.50, 16.80)	20.20 (14.90, 21.00)					
		Min-Max	13.50–25.00	13.00 −23.00					
Median difference			2.50	4.00	-				
Z			−2.94	−2.93					
Standardized effect size			0.63	0.62					
95% CI			(1.70, 4.50)	(2.80, 5.35)					
*p* value^b^			0.003*	0.003*					
Latency (ms)	Fractured	Mean (SD)	2.23 (0.70)	2.49 (1.16)	−0.10	−0.56	0.12	(−0.40, 0.80)	0.61
		Median (IQR)	2.10 (2.00, 2.30)	2.00 (1.70, 4.10)					
		Min-Max	1.40–4.20	1.40–4.40					
	Sound	Mean (SD)	2.24 (0.68)	2.69 (1.15)	0.20	−0.96	0.20	(−0.20, 0.80)	0.37
		Median (IQR)	2.00 (1.80, 2.50)	2.20 (2.00, 4.00)					
		Min-Max	1.60–4.00	1.70–4.80					
Median difference			0.00	0.20	-				
Z			−0.04	−1.71					
Standardized effect size			0.01	0.36					
95% CI			(−0.20, 0.25)	(0.00, 0.45)					
*p* value^b^			0.96	0.09					
Conduction velocity (m/s)	Fractured	Mean (SD)	43.09 (2.12)	43.82 (2.40)	−1.00	−0.56	0.12	(−1.00, 3.00)	0.61
		Median (IQR)	43.00 (41.00, 45.00)	44.00 (42.00, 47.00)					
		Min-Max	40.00–46.00	41.00–49.00					
	Sound	Mean (SD)	44.55 (2.73)	43.73 (3.20)	−1.00	−0.73	0.16	(−4.00, 2.00)	0.48
		Median (IQR)	44.00 (42.00, 47.00)	43.00 (41.00, 47.00)					
		Min-Max	41.00–49.00	40.00–49.00					
Median difference			2.00	0.00	-				
Z			−1.66	−0.04					
Standardized effect size			0.35	0.01					
95% CI			(−0.50, 3.00)	(−2.50, 2.00)					
*p* value ^b^			0.10	0.96					

*µV* Microvolt, *ms* Millisecond, *m/s* Meter per second, *SD* Standard deviation, *IQR* Interquartile range, *Min* Minimum, *Max* Maximum,r rank-biserial correlation effect size**,***CI* Hodges–Lehmann Confidence Interval, Hodges–Lehmann Mean difference

*Statistically significant at *p* < 0.05

^a^Mann-Whitney U test, ^b^Wilcoxon-signed rank

In terms of latency, the mean value for the healthy side at study group was 2.24 ± 0.68 ms and at control group was 2.69 ± 1.15 ms, and that of the fractured side at study group was 2.23 ± 0.70 ms and at control group was 2.49 ± 1.16 ms. No statistically significant difference was detected between healthy and fractured sides in 2 groups in latency (p-value > 0.05). In study group, comparison revealed effect size of 0.01 with CI (−0.20, 0.25) and conventional group showed effect size of 0.36 with CI (0.00, 0.45). These results indicate that nerve latency was stabilized in both groups without significant prolongation (Table 3).

The mean value of conduction velocity for the healthy side at study group was 44.55 ± 2.73 m/s and at control group was 43.73 ± 3.20 m/s, and that of the fractured side at study group was 43.09 ± 2.12 m/s and at control group was 43.82 ± 2.40 m/s. No statistically significant difference was detected between healthy and fractured sides in 2 groups in conduction velocity (p-value > 0.05). In study group, comparison revealed effect size of 0.35 with CI (−0.50, 3.00) and control group showed effect size of 0.01 with CI (−2.50, 2.00). Results indicate that conduction speed was equally preserved in both groups (Table 3).

Regarding post operative pain evaluation, it was decreased significant statistically in all patients in 2 groups along the follow up period. Effect size was high for both customized (0.996) and conventional (0.993) groups, and for CI at 1 st week follow-up was (0.00, 1.00), while at the 4th, 6th, 12th weeks interval, was (0.00, 0.00) indicating a clinical improvement in pain levels. (Tables 3, 4, 5, 6 and 7)

**Table 4:** Difference between customized z plates and conventional 2 miniplates in pain (*N* = 22)

Pain VAS		Customized Z plate *n* = 11	Conventional 2 miniplates *n* = 11	Median difference	Z	Effect size (*r*)	95% CI	*p* value ^a^
Preoperative	Mean (SD)	10.00 (0.00)	10.00 (0.00)	0.00	0.00	0.00	(0.00, 0.00)	1.00
	Median (IQR)	10.00 (10.00, 10.00)	10.00 (10.00, 10.00)					
	Min-Max	10.00–10.00	10.00–10.00					
Postoperative immediate	Mean (SD)	7.27 (0.47)	7.27 (0.47)	0.00	0.00	0.00	(0.00, 0.00)	1.00
	Median (IQR)	7.00 (7.00, 8.00)	7.00 (7.00, 8.00)					
	Min-Max	7.00–8.00	7.00–8.00					
Postoperative (1 week)	Mean (SD)	6.36 (0.50)	6.64 (0.50)	0.00	−1.25	0.27	(0.00, 1.00)	0.30
	Median (IQR)	6.00 (6.00, 7.00)	7.00 (6.00, 7.00)					
	Min-Max	6.00–7.00	6.00–7.00					
Postoperative (4 weeks)	Mean (SD)	3.09 (0.30)	3.18 (0.40)	0.00	−0.61	0.13	(0.00, 0.00)	0.75
	Median (IQR)	3.00 (3.00, 3.00)	3.00 (3.00, 3.00)					
	Min-Max	3.00–4.00	3.00–4.00					
Postoperative (6 weeks)	Mean (SD)	1.00 (0.00)	1.00 (0.00)	0.00	0.00	0.00	(0.00, 0.00)	1.00
	Median (IQR)	1.00 (1.00, 1.00)	1.00 (1.00, 1.00)					
	Min-Max	1.00–1.00	1.00–1.00					
Postoperative (12 weeks)	Mean (SD)	0.00 (0.00)	0.00 (0.00)	0.00	0.00	0.00	(0.00, 0.00)	1.00
	Median (IQR)	0.00 (0.00, 0.00)	0.00 (0.00, 0.00)					
	Min-Max	0.00–0.00	0.00–0.00					
X^2^		54.76	54.63	-				
Effect size (W)		0.996	0.993					
*p* value ^b^		< 0.001*	< 0.001*					

*VAS* Visual Analogue Score, *SD* Standard deviation, *IQR* Interquartile range, *Min* Minimum, *Max* Maximum, *W* Kendall’s w, *r* rank-biserial correlation effect size, *CI* Hodges–Lehmann Confidence Interval, Hodges–Lehmann Mean difference

^a^ Mann-Whitney U test, ^b^ Friedman test

*Statistically significant at *p* < 0.05

**Table 5:** Pairwise comparisons of the difference in pain across time in each type of plates (*N* = 22)

Plate type	Comparison groups		*p* value
Customized Z plate	Preoperative	Postoperative immediate	1.00
		1 week	0.25
		4 weeks	0.003*
		6 weeks	< 0.001*
		12 weeks	< 0.001*
	Postoperative immediate	1 week	1.00
		4 weeks	0.25
		6 weeks	0.004*
		12 weeks	< 0.001*
	1 week	4 weeks	1.00
		6 weeks	0.13
		12 weeks	0.002*
	4 weeks	6 weeks	1.00
		12 weeks	0.18
	6 weeks	12 weeks	1.00
Conventional 2 miniplates	Preoperative	Postoperative immediate	1.00
		1 week	0.34
		4 weeks	0.003*
		6 weeks	< 0.001*
		12 weeks	< 0.001*
	Postoperative immediate	1 week	1.00
		4 weeks	0.34
		6 weeks	0.01*
		12 weeks	< 0.001*
	1 week	4 weeks	1.00
		6 weeks	0.09
		12 weeks	0.001*
	4 weeks	6 weeks	1.00
		12 weeks	0.18
	6 weeks	12 weeks	1.00

Bonferroni post hoc adjustment

*Statistically significant at *p* < 0.05

**Table 6:** Tests of model effects for the linear generalized estimating equation model of the association between pain VAS, type of plate, time, and the interaction between type of plate and time

Explanatory variable	X^2^	df	*p* value
Type of plate	0.26	1.00	0.61
Time	1265.15	1.00	< 0.001*
Time * type of plate	0.15	1.00	0.70

df degree of freedom

*Statistically significant at *p* < 0.05

**Table 7:** Estimated marginal means for the linear generalized estimating equation model of the association between pain VAS, type of plate, time, interaction between type of plate

Explanatory variable			Mean	SE	95% CI
Type of plate	Customized Z plate		4.48	0.09	(4.31, 4.66)
	Conventional 2 miniplates		4.55	0.08	(4.39, 4.70)
Time	Postoperative immediate		7.27	0.09	(7.09, 7.46)
	1 week		3.14	0.07	(2.99, 3.28)
	4 weeks		3.14	0.07	(2.99, 3.28)
Interaction	Customized Z plates*	Postoperative immediate	7.27	0.13	(7.01, 7.54)
		1 week	3.09	0.09	(2.92, 3.26)
		4 weeks	3.09	0.09	(2.92, 3.26)
	Conventional 2 miniplates *	Postoperative immediate	7.27	0.13	(7.01, 7.54)
		1 week	3.18	0.12	(2.95, 3.41)
		4 weeks	3.18	0.12	(2.95, 3.41)

SE Standard Error, CI Confidence interval

**Fig. 5:**
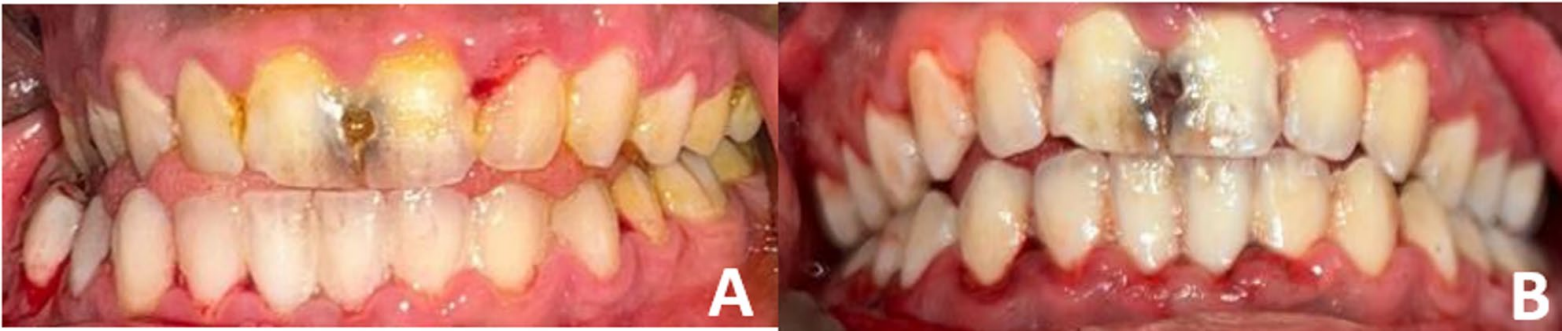
(customized Z plate): **A** Preoperative occlusion. **B** Postoperative occlusion

In terms of occlusion, it was abnormal in both groups preoperatively, while postoperative occlusion, it was normal across the follow up period in 2 groups The effect size was 0.91 for both groups, indicating a consistent surgical outcome in restoring dental relationships (Table 8). (Fig. 5)

**Table 8:** Difference between customized z plates and conventional 2 miniplates in occlusion (*N* = 22)

Occlusion		Customized Z plate *n* = 11	Conventional 2 miniplates *n* = 11
Preoperative	Normal N (%)	0 (0.00)	0 (0.00)
	Abnormal N (%)	11 (100)	11 (100)
Postoperative	Normal N (%)	11 (100)	11 (100)
	Abnormal N (%)	0 (0.00)	0 (0.00)
X^2^		9.09	9.09
Effect size		0.91	0.91
*p* value		0.001*	0.001*

McNemar Exact test

*Statistically significant at *p* < 0.05

**Fig. 6:**
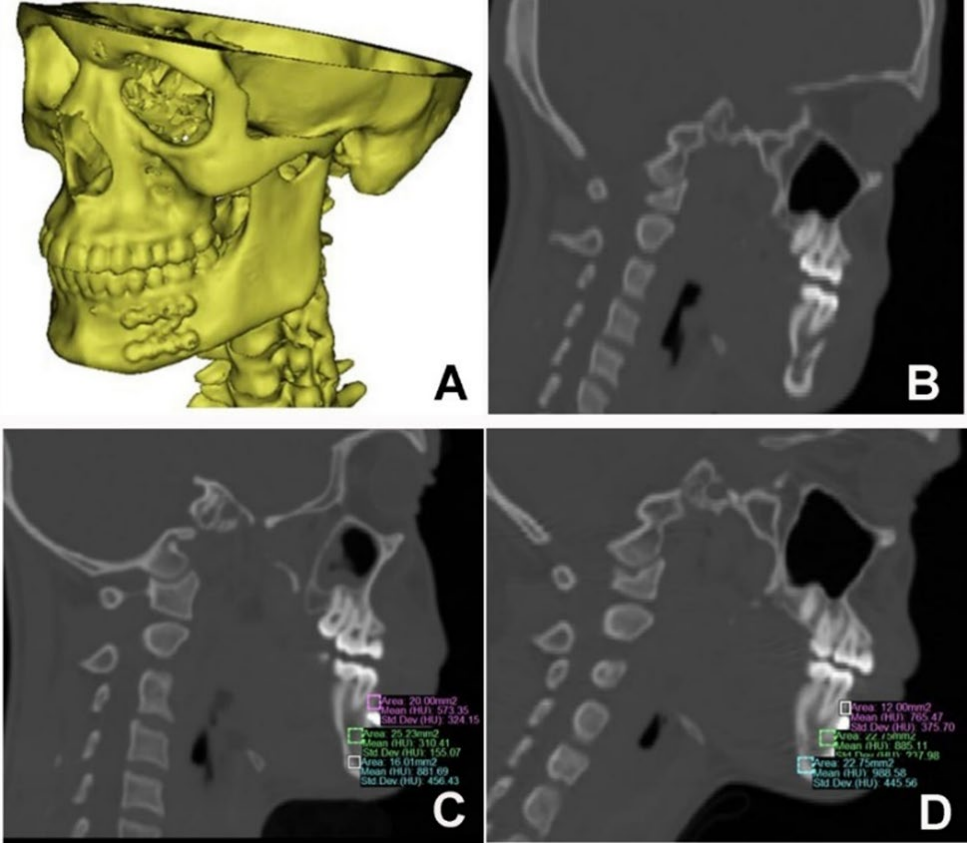
(Study case): **A** 3D reconstruction of mandible treated with customized z miniplate, **B** preoperative CT, **C** measuring bone density at immediate postoperative CT, **D** measuring bone density after 6 months follow up CT

**Fig. 7:**
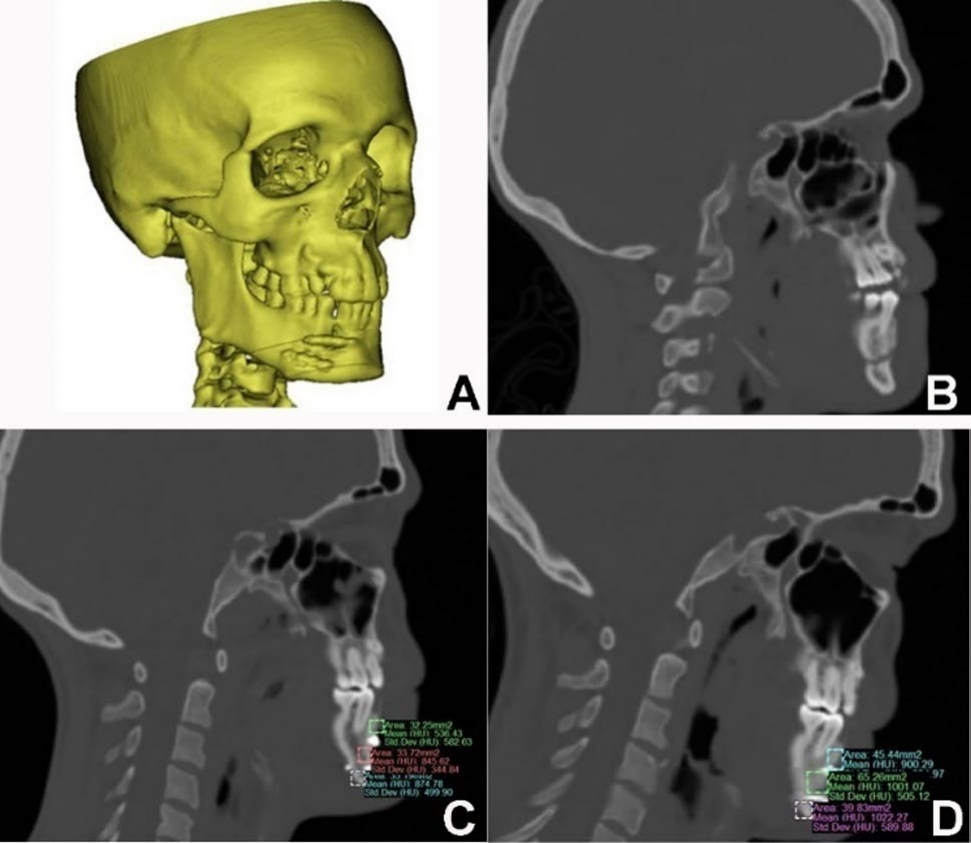
(Control case): **A**) 3D reconstruction of mandible treated with conventional 2 miniplate, **B**) preoperative CT, **C**) measuring bone density at immediate postoperative CT, **D**) measuring bone density after 6 months follow up CT

Regarding the bone density, the immediate post-operative mean at study group was 698.13 ± 59.39 Hounsfield unit (HU) and at control group was 716.27 ± 152.68 Hounsfield unit (HU) which was not significant statistically (*P* = 0.75, effect size: 0.08, CI: −125.63, 141.08), also no detection of statistically significant difference (*P* = 0.61, effect size: 0.12, CI: −132.05, 86.7) after 6 months between study group (992.15 ± 74.06) Hounsfield unit (HU) and at control group (975.61 ± 152.58) Hounsfield unit (HU). Mean bone density overall increased statistically significant along the follow up period for both groups *p* = 0.003 with standardized effect size of 0.62 and CI (−328.50, −262.27) for study and (−322.18, −191.97) for control groups. (Table fTab9) (Figs. 6 and 7).

**Table 9:** Difference between customized z plates and conventional 2 miniplates in bone density immediately postoperative and 6 months postoperative (N=22)

**Bone density** HU		Customized Z plate n = 11	Conventional 2 miniplates n = 11	Median difference	Z	Effect size (r)	95% CI	*p* value ^a^
Immediate postoperative	Mean (SD)	698.13 (59.39)	716.27 (152.68)	26.60	0.36	0.08	(−125.63, 141.08)	0.75
	Median (IQR)	722.14 (660.03, 746.83)	742.58 (582.73, 821.07)					
	Min-Max	562.50–759.02	503.39–968.59					
6 months postoperative	Mean (SD)	992.15 (74.06)	975.61 (152.58)	−30.57	−0.56	0.12	(−132.05, 86.7)	0.61
	Median (IQR)	1002.36 (923.00, 1057.60)	982.28 (811.77, 1095.01)					
	Min-Max	858.29–1105.25	790.95–1276.06					
Median difference		−295.39	−255.25	-				
Z		2.93	2.93					
Standardized effect size		0.62	0.62					
95% CI		(−328.50, −262.27)	(−322.18, −191.97)					
*p* value ^b^		0.003 *	0.003 *					

*HU *Housefield Unit,* SD* Standard deviation, *IQR* Interquartile range, *Min* Minimum, *Max* Maximum, *r* rank-biserial correlation effect size**,***CI* Hodges–Lehmann Confidence Interval, Hodges–Lehmann Mean difference

*Statistically significant at *p*<0.05

^a^Mann-Whitney U test, ^b^Wilcoxon signed-rank

Regarding operation time, in study group, ranged from 30.0 to 65.0 min with a mean (SD) 44.64 ± 11.91 min. In contrast, the control groups showed longer operation time ranged from 60 to 95 min with a mean (SD) 78.64 ± 11.85 min. The statistical analysis detects significant difference (*p* < 0.001) indicating that customized Z miniplate reduce markedly the operation time compared to conventional 2 miniplate. Analysis revealed large effect size (2.86) with CI (−44.57, −23.43), indicating that use of customized Z-miniplates markedly reduced operation time compared to conventional technique (Table 10).

**Table 10:** Difference between customized z plates and conventional 2 miniplates in operation time (*N* = 22)

Operation time (minutes)	Customized Z plate *n* = 11	Conventional 2 miniplates *n* = 11	Mean difference	t	Effect size (d)	95% CI	*p* value
Mean (SD)	44.64 (11.91)	78.64 (11.85)	−34.00	−6.71	2.86	(−44.57, −23.43)	< 0.001*
Median (IQR)	45.00 (35.00, 55.00)	80.00 (65.00, 90.00)					
Min-Max	30.00–65.00	60.00–95.00					

*SD* Standard deviation, *IQR* Interquartile range, *Min* Minimum, *Max* Maximum, *d* Cohen’s d, *CI* Confidence Interval

Independent t-test

*Statistically significant at *p* < 0.05

### Adverse events

Only one case of wound dehiscence reported in each group and were managed by adequate wound debridement, re-suturing and strict instructions were given by maintaining good oral hygiene through frequent tooth brushing, rinsing by mouthwash and prevent any smoking. No other complications were observed. The reported cases of wound dehiscence were considered due to patient adherence to postoperative oral hygiene and unrelated to the fixation hardware (Z-plate or 2 miniplates) and were managed locally. No harms required the discontinuation of the study intervention or further surgical reoperation (Table 11).

**Table 11:** Adverse events assessment summary and treatment at each group

Adverse Events	Customized Z plate *n* = 11	2 miniplates *n* = 11	Note
Infection	0	0	
Wound dehiscence	1	1	*Mild in both cases and managed conservatively without need for reoperation *Healing occurred through follow up visits for both cases
Plate loosening/failure	0	0	
Plate removal	0	0	
Reoperation	0	0	
Malocclusion relapse	0	0	
Radiation related complications	0	0	

Finally, all procedures were completed as planned without intraoperative difficulties, conversions, or plate adjustments were happened in both groups. the need for additional intraoperative modifications.

## Discussion

At this study, pain intensity recorded through (VAS), showed statistically significant decrease across the follow up in 2 groups (*p* < 0.001). This general reduction in postoperative pain was consistent with findings reported by El Nakeeb et al. [25], Agarwal et al. [26] and Melek et al. [27]. Although other studies stated a greater reduction in pain with 3D miniplate due to less operation time [13, 28, 29]. However, we suggest that less pain postoperatively is related to less postoperative interfragmentary mobility that achieved by good stabilization of the fractured parts.

Postoperative occlusion assessment revealed normal occlusion for both groups across the follow up period. This is consistent with other studies on 3D and conventional miniplates [25–27, 30], where minimal statistically significant change in postoperative occlusion across follow up.

The mean bone density along the fracture line increased significantly in both groups after 6 months of follow up (*p* = 0.003). This observed increase in bone density is consistent with previous findings [13, 31] showing significant bone healing through follow up.

Postoperative nerve paresthesia resolved completely across follow up period in both groups. This similar with previous findings [25] reporting total resolution of sensory numbness.

Electrophysiologically, a significant difference was observed in amplitude at the fractured side compared to healthy side in both groups (*p* = 0.003). While mean values of latency and conduction velocity showed no significant difference (*p* > 0.05). This suggests that injury resulted in axonal damage (reflected by decreased amplitude) as opposed to demyelination (reflected by non-significant latency and conduction velocity changes) [32]. Conclusively, while comparison did not reach statistical significance for any electrophysiological measure, these findings should be interpreted with caution due to limited sample. We relied regarding electrophysiological interpretation on relative side-to-side amplitude comparison rather than absolute cutoff values. This approach was adopted due to the significant inter-individual variability in absolute SNAP values and the lack of universally accepted normative data specifically for the mental nerve’s distal latency in the literature. By using the healthy side as an internal control, we ensured a more reliable assessment of axonal injury for each individual patient.

Regarding operation time, customized Z shaped miniplate resulted in a significant decrease compared with conventional 2 miniplates (*p* < 0.001). This is because of better bone adaptation and no intraoperative plate bending a time-consuming step required for conventional fixation and dependent on the surgeon experience [33]. While digital workflow includes preoperative planning and fabrication phase, in our study, this was within the standard 48-hour preoperative time and did not cause any surgical delays. The time invested in digital preparation was effectively offset by the enhanced intraoperative efficiency, making this approach feasible for stable trauma cases where precision is essential. This is similar with Troise et al. [34] who reported comparable mean operational time reduction (from 78.64 ± 11.85 min to 44.64 ± 11.91 min) with customized plates.

The customized Z-miniplate proved practical and easy to use intraoperatively. Surgeons reported easier adaptation with no need to bend and shorter operation time. The open-ended design provided placement with less manipulation and stretching mental nerve and surrounding tissues. This aligns with findings reported by Anchlia S. et al. [35], who used two Orbita plates that achieved optimal healing and minimal mental nerve affection. While both systems showed favorable functional outcomes, the customized Z-plate provides additional practical benefits, particularly the value of patient-specific planning for precise preoperative strategy, reduced intraoperative manipulation, and improved workflow efficiency. However, the need for digital planning and preoperative milling may limit its use in urgent cases.

When comparing our results with the study by D.A. Taalab et al. [36] on patient-specific titanium plates versus conventional miniplates, similar findings were observed regarding shorter operation time and normal occlusion. However, in contrast to their study, which reported significantly lower postoperative pain with customized plates, our analysis found no significant difference in pain levels between the groups at the end of follow-up. This variance highlights that while both customized systems generally improve surgical and functional outcomes, pain results may vary due to differences in specific plate design, surgical technique, or postoperative analgesic management.

In conclusion, statistical analysis of results at present study did not detect any variation between customized Z-shaped miniplate and conventional two-miniplates regarding clinical and radiographical outcomes for parasymphyseal/body mandibular fractures. This is consistent with previous reports that various fixation systems provide clinically acceptable stability. However, due to the limited sample size, these findings should be interpreted with caution and not as evidence of equivalence [30, 37]. 

Regarding adverse events, only one case of wound dehiscence was reported in each group, confirming the low complication rates achieved by both customized and conventional systems, similar to other studies [25, 36]. The single complication may be attributed to issues such as patient compliance or oral hygiene. Finally, while the customized system reduces operative time, the required preoperative CAD/CAM workflow and milling time should be considered, especially in acute cases [17]. The fact that virtual planning was done by the surgical team ensured consistency and efficiency in the workflow.

The findings of this study should be interpreted in the context of several limitations. First, the study design included only unilateral fractures and involved a relatively small sample size, leading to wide 95% confidence intervals and limited statistical power, reflecting a degree of statistical uncertainty. Consequently, findings should be viewed as exploratory and non-significant results should be interpreted with caution. Variables showing no variability across groups were summarized descriptively, which limits comparative statistical evaluation for these outcomes.

Second, bone density assessment was limited only to the fracture site. This perspective, while considered focused, prevents both a whole mandible assessment and comparison with contralateral segment.

Third, the biomechanical behavior of the customized plates was not evaluated, so any statements regarding stabilization or adaptation are based only on clinical observations and should not be interpreted as evidence of biomechanical superiority.

Fourth, the lack of preoperative baseline nerve conduction studies is a limitation in our electrophysiology interpretation. This needs caution, as observed electrophysiological changes cannot be separated into those resulting from initial trauma versus those produced by surgery. Moreover, the absence of bite force measurements is a limitation in fully comparing functional outcomes.

Finally, customized plates involve increased cost and longer preoperative preparation time, which can present a practical limitation. Additionally, the inclusion of patients accepts digital surgery only, although this have favored feasibility of the CAD/CAM application, may limit the overall generalizability (external validity). Radiological blinding during bone density assessment was not feasible due to plate type visibility, and the study also had a relatively short follow-up period.

The retrospective trial registration constitutes an important methodological limitation and a potential source of bias, which should be considered when interpreting the results.

## Conclusion

Although statistical analysis did not detect statistically significant variations between customized Z-shaped miniplates and conventional two-miniplates fixation in terms of postoperative pain, occlusion, mental nerve function and bone density in parasymphyseal/body mandibular fractures, these non-significant results do not necessarily indicate the absence of a true difference, so findings of this study should be interpreted with caution due to limited sample size. The customized Z-miniplate technique showed a significantly shorter operation time, suggesting an intraoperative advantage.

## Supplementary Information

Supplementary Material 1.

## Data Availability

The datasets used and/or analyzed during the current study are available from the corresponding author upon reasonable request.

## Abbreviations

ORIF: Open Reduction and Internal Fixation
CAD/CAM: Computer-aided design and computer-aided manufacturing
CI: Confidence interval
CT: Computed tomography
MMF: Maxillo-Mandibular fixation
NCV: Nerve Conduction Velocity
SNAP: Sensory Nerve Action Potentials
ROI: Region of Interest
HU: Hounsfield units
SD: Standard deviation
VAS: Visual Analogue Scale
SD: Standard deviation
IQR: Interquartile range
Min: Minimum
Max: Maximum
3D: Three-dimensional
ITT: Intention-To-Treat
