# Supplementary material for: Customized z-shaped versus conventional miniplates for fixation of parasymphyseal/body mandibular fractures: a randomized controlled trial evaluating clinical and radiographic outcomes
Source: BMC Oral Health. 2026 Feb 2;26:415. doi: 10.1186/s12903-026-07671-6 (PMC12954998; doi:10.1186/s12903-026-07671-6)
Supplement: Supplementary file 1 — Supplementary Material 1. [file 12903_2026_7671_MOESM1_ESM.docx]

-Regarding the assessment of mental nerve function, the subjective and objective tests revealed significant improvement in sensation across the follow up period for 2 groups p-value <0.001. For the subjective test, the effect size was notably high at 1.00 during the 1^st^ 4^th^ weeks intervals, indicating a strong correlation between time and sensory recovery. Similarly, objective testing demonstrated a consistent recovery pattern with a maximum effect size of 1.00 at 1, 4, 6, and 12 weeks. At the final 12-week follow-up, 100% of patients in both groups achieved complete sensory recovery.

**Table (2’): Difference between customized z plates and conventional 2 miniplates in baseline and early follow up sensory nerve function measured by the subjective and objective tests (N=22)**

| **Sensory nerve function** | | | **Customized Z plate**  **n = 11** | **Conventional 2 miniplates**  **n = 11** | **X^2^** | **Effect size (φ)** | ***p* value ^a^** |
| --- | --- | --- | --- | --- | --- | --- | --- |
| **Subjective test** | **Baseline** | **Yes** **N (%)** | 9 (81.82) | 10 (90.91) | 0.35 | 0.13 | 0.53 |
|  |  | **No** **N (%)** | 2 (18.18) | 1 (9.09) |  |  |  |
|  | **Early follow up** | **Yes** **N (%)** | 8 (72.73) | 8 (72.73) | 0.00 | 0.00 | 1.00 |
|  |  | **No** **N (%)** | 3 (27.27) | 3 (27.27) |  |  |  |
| **X^2^** | | | 0.00 | 0.50 | - | | |
| **Effect size (φ)** | | | 0.77 | 0.52 |  |  |  |
| ***p* value ^b^** | | | 1.00 | 0.50 |  |  |  |
| **Objective test** | **Baseline** | **Yes** **N (%)** | 2 (18.18) | 1 (9.09) | 0.39 | 0.13 | 0.53 |
|  |  | **No** **N (%)** | 9 (81.82) | 10 (90.91) |  |  |  |
|  | **Early follow up** | **Yes** **N (%)** | 9 (81.82) | 9 (81.82) | 0.00 | 0.00 | 1.00 |
|  |  | **No** **N (%)** | 2 (18.18) | 2 (18.18) |  |  |  |
| **X^2^** | | | 5.14 | 4.00 | - | | |
| **Effect size (φ)** | | | 0.22 | 0.52 |  |  |  |
| ***p* value ^b^** | | | 0.02* | 0.04* |  |  |  |

^a^ Chi-squared test with Fisher’s Exact correction, ^b^ McNemar Exact test

*Statistically significant at *p* < 0.05

**Table (2’’): Tests of Model Effects for the Binary Logistic Generalized Estimating Equation Model of the association between postoperative sensory nerve function (measured by subjective and objective tests), type of plate, time, and interaction between type of plate and time (N=22)**

| **Test** | **Explanatory variable** | **X^2^** | **df** | ***p* value** |
| --- | --- | --- | --- | --- |
| **Subjective** | **Type of plate** | 0.15 | 1 | 0.70 |
|  | **Time** | 3.15 | 1 | 0.08 |
|  | **Time * type of plate** | 0.59 | 1 | 0.44 |
| **Objective** | **Type of plate** | 0.19 | 1 | 0.67 |
|  | **Time** | 19.21 | 1 | <0.001* |
|  | **Time * type of plate** | 0.26 | 1 | 0.61 |

df degree of freedom

*Statistically significant at *p* < 0.05

**Table (2’’’): Key Contrasts in the Binary Logistic Generalized Estimating Equation Model of the association between postoperative sensory nerve function (measured by subjective and objective tests) type of plate, time, and interaction between type of plate and time (N=22)**

| **Test** | **Explanatory variable** | | **AOR (95% CI)** | **SE** | ***p* value** |
| --- | --- | --- | --- | --- | --- |
| **Subjective** | **Type of plate** | **A** | 1.00 (0.15, 6.53) | 0.96 | 1.00 |
|  |  | **B** | Reference category | | |
|  | **Time** | **Baseline** | 3.75 (0.63, 22.24) | 0.91 | 0.15 |
|  |  | **Follow up** | Reference category | | |
|  | **Group A* Baseline** | | 0.45 (0.06, 3.45) | 1.04 | 0.44 |
| **Objective** | **Type of plate** | **A** | 1.00 (0.11, 8.73) | 1.11 | 1.00 |
|  |  | **B** | Reference category | | |
|  | **Time** | **Baseline** | 0.02 (0.002, 0.24) | 1.21 | 0.002* |
|  |  | **Follow up** | Reference category | | |
|  | **Group A* Baseline** | | 2.22 (0.22, 46.80) | 1.55 | 0.61 |

AOR Adjusted Odds Ratio, SE Standard Error, CI Confidence interval

*Statistically significant at *p* < 0.05
